# Pathogenesis of Aging and Age-related Comorbidities in People with HIV: Highlights from the HIV ACTION Workshop

**DOI:** 10.20411/pai.v5i1.365

**Published:** 2020-06-17

**Authors:** Dana Gabuzda, Beth D. Jamieson, Ronald G. Collman, Michael M. Lederman, Tricia H. Burdo, Steven G. Deeks, Dirk P. Dittmer, Howard S. Fox, Nicholas T. Funderburg, Savita G. Pahwa, Ivona Pandrea, Cara C. Wilson, Peter W. Hunt

**Affiliations:** 1 Department of Cancer Immunology and Virology; Dana-Farber Cancer Institute; Boston, Massachusetts; Department of Neurology; Harvard Medical School; Boston, Massachusetts; 2 Department of Medicine; David Geffen School of Medicine; University of California; Los Angeles, California; 3 Department of Medicine; University of Pennsylvania School of Medicine; Philadelphia, Pennsylvania; 4 Department of Medicine; Case Western Reserve University School of Medicine; Cleveland, Ohio; 5 Department of Neuroscience; Lewis Katz School of Medicine; Temple University; Philadelphia, Pennsylvania; 6 Department of Medicine; University of California; San Francisco, California; 7 Department of Microbiology and Immunology; University of North Carolina School of Medicine; Chapel Hill, North Carolina; 8 Department of Pharmacology and Experimental Neuroscience; University of Nebraska Medical Center; Omaha, Nebraska; 9 Division of Medical Laboratory Science; School of Health and Rehabilitation Sciences; Ohio State University College of Medicine; Columbus, Ohio; 10 Department of Microbiology and Immunology; University of Miami Miller School of Medicine; Miami, Florida; 11 Department of Microbiology and Molecular Genetics; School of Medicine; University of Pittsburgh; Pittsburgh, Pennsylvania; 12 Department of Medicine; Division of Infectious Diseases; University of Colorado Anschutz Medical Campus; Aurora, Colorado

**Keywords:** HIV, aging, inflammaging, cellular senescence, microbiome

## Abstract

People with HIV (PWH) experience accentuated biological aging, as defined by markers of inflammation, immune dysfunction, and the epigenetic clock. They also have an elevated risk of multiple age-associated comorbidities. To discuss current knowledge, research gaps, and priorities in aging and age-related comorbidities in treated HIV infection, the NIH program staff organized a workshop held in Bethesda, Maryland in September 2019. This review article describes highlights of discussions led by the Pathogenesis/Basic Science Research working group that focused on three high priority topics: immunopathogenesis; the microbiome/virome; and aging and senescence. We summarize knowledge in these fields and describe key questions for research on the pathogenesis of aging and age-related comorbidities in PWH. Understanding the drivers and mechanisms underlying accentuated biological aging is a high priority that will help identify potential therapeutic targets to improve healthspan in older PWH.

## INTRODUCTION

**Figure 1.**
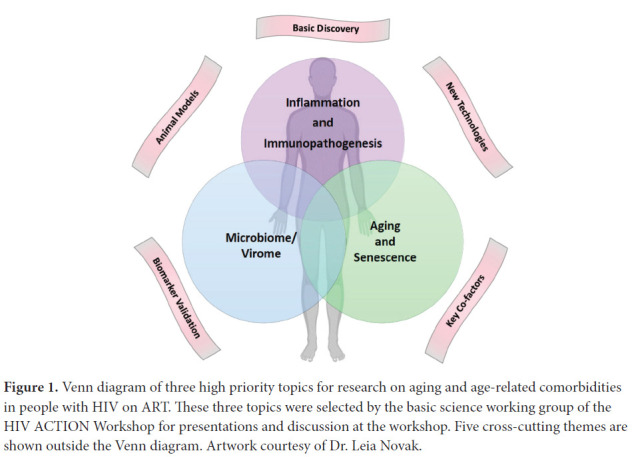
Venn diagram of three high priority topics for research on aging and age-related comorbidities in people with HIV on ART. These three topics were selected by the basic science working group of the HIV ACTION Workshop for presentations and discussion at the workshop. Five cross-cutting themes are shown outside the Venn diagram. Artwork courtesy of Dr. Leia Novak.

Despite the success of combination antiretroviral therapy (ART) in achieving durable virologic suppression, people with HIV (PWH) are at increased risk for multiple comorbidities associated with aging in the general population, including cardiovascular disease (CVD), lung disease, liver disease, kidney disease, diabetes, neurocognitive disorders, decreased bone mineral density, malignancies, and other diseases [1]. Evidence suggests that treated HIV infection is associated with accentuated aging phenotypes, and these age-related comorbidities can occur at younger ages [2, 3]. The pathophysiologic mechanisms that drive biological aging and age-related comorbidities are not well understood, and the impact of HIV and ART on the biology of aging remains poorly defined. To discuss current knowledge, research gaps, and priorities in HIV-associated comorbidities, a pan-NIH workshop on HIV-associated Comorbidities, Coinfections, and Complications (HIV ACTION) was held in Bethesda, Maryland on September 19-20, 2019. Six working groups were convened and met over the course of a year to prepare for the workshop. This article describes highlights from the planning summaries, talks, and discussions led by the Pathogenesis/Basic Science Research working group, which focused on the following three high priority topics selected by working group members: 1) Immunopathogenesis; 2) Microbiome/Virome; and 3) Aging and Senescence (Figure 1). Here, we summarize the background, rationale, and key questions for research on the pathogenesis of aging and age-related comorbidities in PWH, and how basic research on these questions might lead to the discovery of new targets for prevention and therapeutic intervention.

### Immunopathogenesis and HIV-associated comorbidities

#### Immune activation and inflammation

Levels of immune activation and inflammation remain elevated in PWH on ART, even when viral suppression has been maintained for several years, and may contribute to morbidity and mortality [4, 5]. Key questions for basic research on immune activation/inflammation and immunopathogenesis related to development of HIV-associated comorbidities are shown in Table 1. Persistent inflammation likely contributes to multiple end-organ diseases in PWH, including CVD, liver, and kidney diseases, neurocognitive disorders, malignancies, and other diseases. While specific mechanisms that underlie each of these comorbidities may involve both shared and unique pathways, biomarkers of immune activation and inflammation (ie, IL-6, sCD14, sCD163, D-dimer, soluble TNF receptors 1 and 2) are broadly associated with development and progression of these comorbidities in PWH [4, 5]. Inflammatory mediators are also linked to development of these comorbidities with aging in uninfected populations. Although definitive evidence establishing a causal role of inflammation in driving disease risk in treated HIV infection is lacking, several lines of evidence support this possibility. A recent clinical trial of the IL-1β inhibitor canakinumab in HIV-uninfected individuals with heart disease showed that reducing inflammation decreases cardiovascular events and mortality from cancer [6]. Furthermore, comparative studies between progressive and non-progressive SIV infections suggest that SIV-induced inflammation contributes to development of CVD and other comorbidities [7]. Persistent inflammation and “inflammaging” (increased inflammation with advancing age) have also been identified as likely contributors to frailty [8, 9], a geriatric phenotype related to physical aging. Whether inflammatory profiles associated with increased risk of age-related comorbidities differ between PWH and uninfected populations, and whether their potential mediators and pathways are similar or different, is still unclear.

**Table 1.** Key Questions for Basic Research on Immunopathogenesis in HIV-associated Comorbidities

**Key Questions**
**What are the drivers of chronic innate and adaptive immune activation in PWH?** How do inflammatory profiles of untreated HIV, treated HIV, and comorbidities in the general population differ—or are they superimposable? Are drivers of comorbidities in PWH and the aging general population similar—or do different inflammatory pathways drive each towards development of end-organ disease? What is the impact of exposure to microbial elements, inflammatory lipids, low-level HIV replication or expression, co-pathogens such as CMV, EBV, HHV-8, HCV, and other human viruses, and inflammatory cytokines that may drive activation, expansion, dysfunction, and altered trafficking/retention of immune cells in tissues? Which inflammatory mediators drive pathology—and which are only markers of immune activation/inflammation? How do other exposures (eg, recreational drugs, tobacco, alcohol, etc) affect immune activation and inflammation? What are the immune cell and other inflammatory cell subsets with abnormal functions, and how do they contribute to residual immune activation/inflammation and comorbidities during treated HIV infection?
**Is residual immune activation/inflammation similar in women and men and, if not, what are the pathways that differ?**
**Do HIV and ART induce defects in hematopoiesis and tissue sites that ultimately drive chronic immune dysfunction in PWH? For example:** Tissue sites/reservoirs (lymph node, gut, brain, etc) with residual virus expression that drive immune activation/inflammation under ART? Effects of ART regimens on innate and adaptive defense mechanisms? Effects of early ART on hematopoiesis, gut, and other organ dysfunctions that could impact inflammatory pathways? Can we design studies in uninfected individuals initiating ART as PrEP to untangle the metabolic, neurological, bone, and inflammatory consequences of some ART drugs? Effects of ART on hematopoietic malignancies that are severely overrepresented in PWH?
**Can we develop or use tools to predict or identify specific disease pathology?** Systemic profiles of soluble and cellular markers of inflammation and coagulation linked to progression of specific disease pathologies? Imaging methods that accurately define vascular pathology, neurocognitive impairment, and metabolic diseases including non-alcoholic fatty liver disease (NAFLD)/non-alcoholic steatohepatitis (NASH)? NHP models to define disease pathology and mechanisms of immunopathogenesis in SIV infection that reflect immunopathogenesis in HIV infection?
**Can we develop therapeutics to reduce immune activation/inflammation and what should be the main targets for such interventions?** Should these therapies target elements common to multiple inflammatory pathways activated in HIV disease or should we specifically target more upstream drivers of inflammation, such as specific co-pathogens or inflammatory lipids? How will immune-targeted interventions affect host defenses against pathogens, autoimmunity, and malignancy? How will these interventions affect immune homeostasis?

Several potential drivers of chronic inflammation in PWH have been identified, including low-level residual HIV expression during suppressive ART, microbial dysbiosis and translocation, coinfections (eg, cytomegalovirus [CMV], Epstein Barr Virus [EBV], human herpesvirus virus 8 [HHV-8], Hepatitis C virus [HCV]), altered lipid profiles (eg, elevated cholesterol and/or triglycerides, inflammatory oxidized lipids), and lifestyle factors. Each of these potential drivers has been linked to comorbidities in PWH [4, 10]. Lifestyle factors such as tobacco smoking, recreational drug use, poor diet, and lack of physical activity along with genetics and coinfections (particularly CMV) have all been linked to low-grade inflammation and development of age-related diseases in the general population (ie, CVD, diabetes, liver disease, age-related cognitive decline). Importantly, many of these lifestyle factors are enriched among PWH [11–14]. Residual immune activation and inflammation, and some other key drivers, may differ between men and women [15], racial/ethnic groups [16], and geographic regions [17] and are likely to be influenced by genetic and environmental factors as well. The list of candidate drivers described here is likely incomplete and requires further study in PWH and uninfected populations to identify root drivers and their relative importance in different settings. To address these questions, animal models are excellent biological systems in which strictly controlled studies can be performed to dissect relative contributions of different factors that are difficult to study in humans. A related question is understanding the distinct patterns of chronic inflammation (ie, “flavors of inflammation”) that underlie different organ system comorbidities in different settings. However, not all age-related morbidities are increased by HIV infection (eg, prostate, colon, and breast cancer). Additionally, PWH who start HIV treatment at high CD4 counts may be protected from some morbidities (eg, cardiovascular disease, neurocognitive dysfunction), but remain at abnormally high risk for infections and infection-related malignancies [18]. Understanding the inflammatory pathways responsible for infectious vs non-infectious complications, and the degree to which they persist in individuals who start ART early vs late in the disease course, will be important to identify the most appropriate interventional targets for different settings.

#### Immune cell populations and trafficking

A key issue in immunopathogenesis studies is understanding the effects of HIV and ART on immune cell subsets and their relation to residual immune activation, inflammation, and age-related comorbidities in treated HIV infection. Dysregulation of the immune system due to HIV itself, coinfections, or bystander mediators of inflammation may drive inappropriate activation and retention of immune cells within tissue sites such as blood vessels, liver, adipose tissues, and the central nervous system (CNS). Circulating immune cell populations that include activated monocytes expressing pro-coagulants (eg, tissue factor), as well as increased numbers of mature activated CD8^+^ T cells that can home to endothelial surfaces via expression of homing receptors including the fractalkine receptor (CX3CR1), lymphocyte function-associated antigen 1 (LFA-1), macrophage-1 antigen (Mac-1) [19, 20], C-C chemokine receptor type 2 (CCR2), and type 5 (CCR5) [21], are detected in PWH, and may be linked to development of CVD and other comorbidities [22–26]. Altered migration of activated immune cells to, and retention within, these tissues may also influence development of other end-organ diseases. Additionally, T cell subsets may be inappropriately retained within lymph nodes of PWH [27], potentially contributing to increased lymph node inflammation, fibrosis, and failure of immune cell reconstitution [28, 29]. Some ART drugs may have effects on innate and adaptive defense mechanisms, as well as on hematopoiesis, gut, and other organ dysfunction, which could also influence development of comorbidities. Chemokine receptor antagonists, in particular, may modulate migration of immune cells to the liver [30], CNS [31], and gut-associated lymphoid tissues (GALT) [32], potentially modulating immune activation and end-organ disease. It is important to determine whether HIV and ART induce defects in hematopoiesis that have downstream effects on immune activation, inflammation, and immune dysfunction [33]. Additionally, it will be important to identify the tissue sites/reservoirs (lymph node, gut, brain, etc) responsible for residual virus production that could drive immune activation/inflammation under ART [34]. For such studies, animal models are useful because they allow access to a variety of tissues and use of tagged virus strains that enable detailed characterization of the tissue sites harboring residual virus. Studies involving more limited tissue sampling of PWH will also be important, as the degree of viral suppression in most animal models may not fully reflect the degree of viral suppression in tissues among PWH on long-term ART [35]. Identification of unique biomarker signatures and immune cell populations that predict comorbid conditions will provide insights into mechanisms that drive specific end-organ diseases, and may identify populations that could benefit from personalized prevention and therapeutic strategies.

#### Targeting upstream drivers of immune activation/inflammation

Whether disease-associated inflammatory profiles differ in aging PWH and uninfected populations is an important question for development of targeted therapeutic interventions. Although ART prolongs the lifespan of PWH, the quality of life is dampened by side effects of ART including metabolic diseases, decreased bone mineral density, and alterations in lipid profiles and mitochondrial function [36, 37]. Therefore, understanding the effects of ART on the host is important for the optimal design of new strategies to prevent morbidity. It is unclear whether targeting common elements shared by multiple immunopathogenic pathways in treated HIV infection, targeting upstream drivers of activation/inflammation, or direct targeting of the viral reservoir itself will be most effective. It is also unknown whether comorbidities associated with treated HIV infection are driven by overlapping or distinct immunological mechanisms. Studies in uninfected individuals initiating ART as pre-exposure prophylaxis (PrEP) and in uninfected nonhuman primate (NHP) models will be important to untangle the metabolic, neurological, bone, and inflammatory consequences of some ART drugs. Clinical intervention trials offer unique opportunities to concurrently test a mechanistic hypothesis and explore potential clinical utility of the intervention, novel readouts, and disease models. Animal models also provide great opportunities to probe interventional strategies, particularly those that have safety concerns or require extensive tissue sampling. For these interventional approaches, it is important to evaluate not just the pathway being targeted, but also parallel inflammatory pathways and/or root drivers that might be affected in either a positive or negative way (ie, the “whack-a-mole” problem) [38]. Addressing these questions will help to define determinants of end-organ disease risk in PWH, targets for intervention, and whether morbid complications of aging in the general population share common pathways with these same outcomes in PWH.

### Microbiome/virome in HIV-associated comorbidities

#### HIV-associated changes in the microbiome

The microbiome consists of the entirety of bacteria, fungi, and viruses that live in concert with the human body, including the gut, respiratory tract, female and male genital tract, oral cavity, skin, and other sites. Among these, the gut microbiome has received the greatest attention. Given the link between inflammation and comorbidities, two observations underlie high interest in the microbiome in HIV infection: (a) the gut microbiome plays a key role in both systemic and local mucosal immunological development and regulation [39–41]; and (b) profound injury to the gut immunological barrier occurs very early in HIV infection, is incompletely repaired with ART, and enables systemic translocation of microbial products that contribute to inflammation [42–45]. Research outside the field of HIV is revealing deep connections between the microbiome, host immunity [46], and function of multiple organ systems, in which dysbiosis is linked to CVD [47], liver disease [48], neurological conditions [49], cancer [50, 51], and other diseases. Metabolome alterations in plasma and other sites may reflect not only microbial products, but also secondary effects of the microbiome on host-derived metabolites mediated through its effects on bile acids, host enzymes, and local mucosal inflammation [52, 53]. Key questions for research on the micro-biome that are relevant for the development of HIV-associated comorbidities are shown in Table 2.

**Table 2.** Key Questions for Basic Research on the Microbiome/Virome in HIV-associated Comorbidities

**Key Questions**
**Does the microbiome (bacterial, viral, fungal) play a contributory role in HIV comorbidities?** Are microbiome changes during HIV infection mainly a consequence of gut epithelial or immune damage, or are they a contributor to, or perpetuator of, this damage, microbial translocation, and/or systemic inflammation and comorbidities? Are changes in the virome (gut, plasma, oral, vaginal) contributors to systemic inflammation and/or end-organ comorbidities, or markers of immunological or barrier dysfunction? How do HIV-associated microbiome alterations at mucosal sites beyond the gut (eg, lung, oral) affect comorbidities such as cancer, chronic lung disease, CVD or CNS disease, or others?
**How do other cofactors interact with HIV infection to affect the microbiome and comorbidi-ties?** Do ART, geography, diet, smoking, sexual behavior, gender, and other factors impact the microbiome and its function in HIV-associated comorbidities? How do coinfections, particularly those that are prevalent in resource-poor areas and may be considered part of the “microbiome” in those areas (eg, candida, GI helminths, malaria, EBV, HHV-8) impact comorbidities directly or via effects on the more conventional gut microbiome?
**What are the mechanisms of host/HIV/microbiome interactions?** What are the responsible mediators (protein, carbohydrate, small-molecule metabolites, etc), and how do microbial products influence the host metabolome and vice-versa? How does ART impact these pathways? Beyond taxonomy, what functional changes to the microbiome are involved in these mechanisms?
**Can the microbiome in HIV be manipulated to modify these pathways-via replacement, microbe targeting, microbe-directed small-molecule therapeutics, or other approaches? Would such approaches also need to target injured mucosa to achieve sustained impact?**

Early reports showed gut microbiome differences in MSM with HIV compared to healthy non-MSM controls [54–57], with subsequent studies clarifying that the microbiome is affected both by HIV infection and by sexual behavior [58–61]. Other factors known to affect the microbiome include geography, diet, coinfections, and age [62, 63], while further research is needed on the effects of ART itself, smoking, gender, etc. While the changes seen in PWH vary between studies, a common theme includes decreased diversity and relative enrichment of certain taxa and depletion of others [61]. Several organisms enriched in HIV-associated gut microbiomes have proinflammatory properties [64]. The degree of dysbiosis is also linked to immune status and systemic inflammation [65–68]. Coinfections, particularly those that are prevalent in resource-poor areas and may also be considered part of the “microbiome” in those geographic regions (eg, candida, GI helminths, malaria, EBV, HHV-8), are linked to comorbidities, vaccine response, and HIV transmission either directly or via effects on the conventional gut microbiome. Fecal metabolome profiles are also altered, including tryptophan and bile acid metabolism [55, 69, 70]. In the SIV macaque model of AIDS, marked expansion of the enteric virome has been reported [71], with some changes also identified in PWH [57, 59].

The microbiome has been implicated as a contributing factor to CVD in the general population, via metabolism of dietary nutrients by gut microbiota to trimethylamine (TMA), which is converted to trimethylamine N-oxide (TMAO), which has direct effects on the heart and vasculature as well as kidney and other organs [72, 73]. Other microbiome-related pathways have been implicated in animal models and human populations in gastrointestinal, liver [48], neurobehavioral [49], metabolic, neoplastic [50, 51], and other diseases. Many of these findings involve pathways mediated by systemic inflammation and/or microbial metabolite alterations, although local microbiome populations have also been implicated in development of cancers and lung disease [74–76]. The microbiome is a key determinant of the integrity and function of the gut mucosal barrier [77, 78], and is a major target of therapeutics in inflammatory bowel disease [79, 80]. In HIV infection, alterations in the microbiome and metabolites that are affected by microbes, such as TMAO and kynurenines, have been linked to CVD and liver disease [55, 81–83], although not all studies agree [84]. Some of these pathways have also been linked to neurocognitive or psychiatric comorbidities [85–87]. ART can alter some of these microbiome-related pathways [88–90]. However, it remains to be determined whether microbiome changes identified in HIV infection are cause or consequence. Are they simply a reflection of gut epithelial or immune damage, or do they contribute to or perpetuate such damage, systemic inflammation, and comorbidities?

Because the field of microbiome research is relatively new, the working group considered the overarching question of whether research on microbiome/HIV comorbidities should be carried out contemporaneously with research on related comorbidities outside of the HIV context. While this discussion is focused on HIV comorbidities, the close link between comorbidities and inflammation, the microbiome's role in the development and regulation of immune function, and links between inflammation and immunopathogenesis means that many of the questions raised here are pertinent to whether the microbiome impacts immunopathogenesis, including immune recovery following ART. The microbiome may also be important in other key aspects of HIV disease apart from comorbidities, such as transmission (through the female and male genital tracts [91, 92]), prevention (vaccine efficacy affected by gut microbiome [93, 94], and microbicide activity affected by vaginal microbiome [95]). With recent evidence that the microbiome can modulate the effects of immunotherapy in cancer [96–98], it is reasonable to ask whether microbial populations will determine responses to immunotherapy targeting morbidity and HIV eradication.

### Microbiome as potential therapeutic target

Interventional studies have not yet demonstrated a clear impact of the microbiome on systemic inflammation in PWH. For example, a recent study of the probiotic Visbiome failed to find alterations in any systemic markers of immune activation in treated HIV infection [99]. Host metabolome profiles appear to differ in PWH compared to those in uninfected people, and some of these differences may determine the risk of some comorbidities. Thus, identifying the key micro-biome-related pathways that could most strongly drive disease risk (and how best to alter them) has the potential to improve the effectiveness of targeted microbial-directed therapy. Given rapid progress in research on microbiome-organ interactions and health consequences, focused efforts in the context of HIV can leverage that progress to accelerate discoveries that might ameliorate comorbidities. Incorporating microbiome/virome/fungome and metabolome measurements into comorbidity-focused HIV clinical trials could provide a platform that will help to advance such discovery.

Microbiome research in animal models has been valuable in fields outside of HIV (eg, inflammatory bowel disease, diabetes, obesity, mood/anxiety disorders, etc). SIV-infected NHPs are the best model for HIV pathogenesis, but these models are logistically more challenging and costly than small animal models, and the human and NHP microbiomes have important differences. Small animal models for studies of the microbiome that are more reflective of the human condition are important for defining microbiome-organ interactions in health and disease. Thus, a mix of small animal, NHP, and human studies are needed to advance the field, and to discover, develop, and model interactions between the microbiome and comorbidities. Further studies are also needed to determine whether the microbiome can be manipulated to modify these pathways—via replacement, microbe targeting, microbe-directed small molecule therapeutics, or other approaches—and whether such approaches also need to target the injured mucosa to achieve sustained impact on immune function and disease outcomes.

### Aging and Cellular Senescence in HIV

#### Is aging accentuated or accelerated in HIV?

Despite the positive effects of ART on longevity, lifespans of PWH may still fall short of the general population's [100] and is characterized by an increased risk for earlier development of age-associated comorbidities [101–103]. Aging can manifest at the level of inflammation, immune response, and cellular senescence in any organ system. Additionally, aging affects both immune surveillance of cancer cells and cell-intrinsic DNA integrity, leading to an exponential increase in malignancies with age.

**Figure 2.**
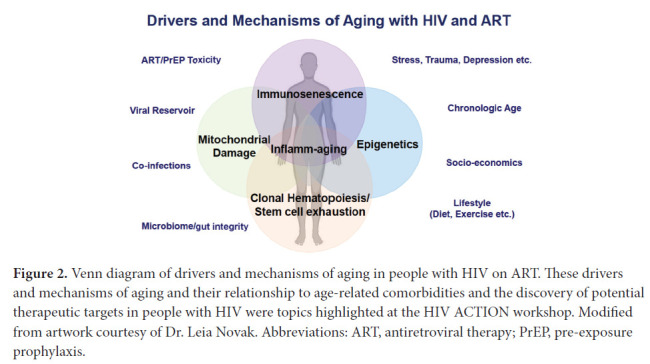
Venn diagram of drivers and mechanisms of aging in people with HIV on ART. These drivers and mechanisms of aging and their relationship to age-related comorbidities and the discovery of potential therapeutic targets in people with HIV were topics highlighted at the HIV ACTION workshop. Modified from artwork courtesy of Dr. Leia Novak. Abbreviations: ART, antiretroviral therapy; PrEP, pre-exposure prophylaxis.

PWH exhibit declines, at a significantly younger age, in gait speed [104] and grip strength [105], consistent with increased incidence of frailty-related phenotypes [106] and frailty [107]. In addition to HIV and ART, other factors that may affect biological aging in PWH include socio-demographic, socioeconomic, and/or behavioral factors (Figure 2) [108]. While HIV does not necessarily increase the rate at which these complications increase with advancing age (ie, accelerated aging), the higher risk of age-related multi-morbidity and functional decline at any given age among PWH is consistent with an accentuated aging model [109].

Treated HIV infection, like aging, is associated with increased inflammatory and coagulation markers which, in turn, are associated with increased risk of many aging-related comorbidities and mortality [110]. Nevertheless, immunologic predictors of age-related complications may be different in people with and without HIV. For example, the magnitude of the IL-2 response by CD4^+^ T cells in response to stimulation by CMV peptides predicts frailty onset in uninfected men, but fails to do so in men with HIV [111]. Similarly, CD8+ T cell phenotypes associated with immunosenescence (CD28-CD57+) predict mortality in HIV-uninfected elderly individuals, but not in PWH [112, 113]. Higher rates of tobacco smoking, recreational drug use, depression, stigma, and trauma in PWH may also contribute to observed differences in people with versus without HIV [114]. Despite the difficulty in assessing whether HIV infection accelerates or accentuates aging, the number of people aging with HIV infection continues to increase; therefore, understanding these interactions is critical to the rational design of therapies to increase both the healthspan and lifespan of PWH as they age.

Key questions for research on aging and cellular senescence in relation to development of HIV-associated comorbidities are shown in Table 3. Discussions focused on whether the etiology of aging-related inflammation differs between PWH and uninfected individuals, and how “inflammaging” is influenced by biological sex, as well as by coinfections, lifestyle, sociodemographic/socioeconomic status, neuropsychiatric conditions (depression, addictions, etc), and/or behavioral factors. The deleterious influence of inflammation and immune activation on immune function as evaluated by vaccine responses in the context of age and HIV infection was discussed [115–118]. Other discussions included the influence of ART on drivers of inflammation, and whether newer, current ART regimens contribute to the inflammatory environment through mitochondrial toxicities or other mechanisms. Evidence suggests that some non-reversible pretreatment pathologies (eg, lymphoid fibrosis, GALT disruption) [119], along with CMV or other coinfections (eg, HCV), contribute to inflammation and age-associated comorbidities of aging [120, 121]. However, it remains unclear whether these factors are causal or only associated.

**Table 3.** Key Questions for Basic Research on Aging and Senescence in HIV Infection

**Key Questions**
**Do the mechanisms and mediators of age-related end-organ diseases (ie, sarcopenia, neuro-cognitive dysfunction, malignancies, etc) differ in aging with and without HIV and ART?** How are inflammatory pathways and their root causes similar or different with regard to their profiles and contributions to age-related comorbidities in the presence or absence of HIV? Does the etiology of aging-related inflammation differ between PWH and uninfected individuals? How is it influenced by gender, coinfections, lifestyle, sociodemographic and socioeconomic status, neuropsychiatric conditions (depression, addiction, etc), and/or behavioral factors? How does ART influence drivers of inflammation, and does it introduce any new sources or exacerbate existing ones? To what extent do newer current ART regimens in-duce mitochondrial toxicities and contribute to the inflammatory milieu after ART initiation? How do non-reversible pretreatment alterations (eg, lymphoid fibrosis, GALT disruption) contribute to inflammation and comorbidities of aging? What is the role of CMV or other infections (eg, HCV, EBV, HHV-8) in inflammation and comorbidities of aging? Is it causal or only correlated? How do neuropsychiatric conditions over-represented in PWH (eg, depression and addiction) contribute to inflammaging and comorbidities?
**Why are senescent, terminally differentiated and activated T cells, particularly CD8+ T cells, associated with comorbidities in the elderly and with accelerated epigenetic aging in HIV infection? Are they causative, contributory, or only correlative? Would senolytics change this landscape in the presence or absence of HIV?** Is inflammation the major driving force behind the accelerated epigenetic aging observed in PWH, and if so, what underlying mechanism(s) elicits these changes?
**Can we identify lifestyle, behavioral, and environmental variables that positively modify the epigenetic, mitochondrial, and inflammatory background in successfully treated PWH, and do these variables differ in their impact in PWH vs uninfected adults in mitigating morbidities and mortality?** What are the long-term consequences of an accelerated aging epigenome in PWH? Using epigenetics, can we find genes or intersecting pathways that are prognostic for morbidities/mortality, thus allowing—not only for discovery of biomarkers of impending disease and death—but for novel pharmaceutical intervention strategies?
**What role does damage to critical hematopoietic stem cell niches and lymphoid microenvironments by HIV and ART play in age-related comorbidities, especially younger onset of malignancies and their more aggressive clinical presentation?**

### Evidence of premature aging from epigenetic clock studies

Studies of the “epigenetic clock” provide evidence suggesting greater advancement of biological aging in PWH. In particular, untreated HIV infection results in an average advancement of DNA methylation age in peripheral blood mononuclear cells (PBMC) of ~8.4 years or more according to the original “epigenetic clock” and ~5.9 years by the newer GrimAge epigenetic clock, which predicts morbidity and mortality [122]. Non-aging related genes are also epigenetically modified, potentially pointing towards unique insults that HIV infection might bring to the development of morbidities and mortality. However, these epigenetic changes and their biological significance remain largely unexplored. Two years of ART treatment only moderately readjust the PBMC epi-genetic clock to more age-appropriate patterns. For example, one study found that ART-treated individuals may remain epigenetically older by ~5 years for even longer than two years post-ART [122, 123]. Studies looking at longer ART duration, timing of ART initiation, age at infection and ART initiation, and identifying new potentially deleterious epigenetic changes introduced by ART are still needed. Additionally, newer epigenetic clocks are being tested in uninfected populations to predict morbidity and mortality and to identify modifiers that might decrease the risk of these outcomes.

Is inflammation the major driving force behind the accelerated epigenetic clock observed in PWH, and if so, what are the underlying mechanisms that elicit these changes? To understand how biological aging influences age-related comorbidities in older PWH, it will be important to evaluate epigenetic changes in relevant tissues and in specific immune cell types, as the inflammatory milieu may differ by site, organ, and cell type. Knowledge gained from analyses of these epigenetic changes in specific cell types and organs can then be leveraged to address the above questions and potentially accelerate intervention research.

The utility of these epigenetic changes as a biomarker for efficacy of treatment is uncertain, as there is no evidence that reversing these epigenetic changes is associated with better outcomes, but it warrants further study. Specific epigenetic changes unique to HIV have been identified [123]. These epigenetic changes could then be evaluated to determine whether an intervention can alter health outcomes, long-term consequences of epigenetic aging markers in PWH, and genes or intersecting pathways that are prognostic for morbidities and mortality. This approach might allow not only for discovery of biomarkers of impending disease and death, but also for discovery of novel intervention strategies.

### Aging, senescence, inflammation, and age-related comorbidities

During aging in the general population, as the risk of morbidities and mortality increases, lifestyle factors, psychological stress, mental health, environmental toxins, chronic infections such as CMV, and a “leaky” gut can set up a background of inflammation that increases with advancing age (inflammaging) mediated through oxidative stress, mitochondrial damage (mtDNA, formylated peptides), and cellular activation/proliferation [124, 125]. Many of these mechanisms also drive induction of senescent cells [126], with a senescence-associated secretory phenotype [SASP] [127] that in turn can elaborate proinflammatory molecules and exacerbate inflammaging [128]. SASP of senescent cells can thus promote the development of comorbid conditions such as insulin resistance and CVD [129]. A multitude of factors listed above has been implicated in acquisition of SASP by senescent cells; in addition to DNA damage, telomeric dysfunction, and epigenetic changes, these factors also include social stress, lifestyle, environment, and chronic infections. These concepts are discussed in the field of geroscience, which links aging to chronic diseases [130, 131]. Senescent, terminally differentiated, and activated T cells, particularly CD8^+^ T cells, are associated with morbidity and mortality in the elderly.

In PWH, accumulation of senescent cells is associated with advancement of epigenetic aging, though it is unclear whether they play a causative or contributory role or are only correlative. Other contributing factors that may promote biological aging and age-related comorbidities in HIV include antigen-driven proliferation, shortening of telomeres, accumulation of DNA damage, disruption of the stem cell niche, and stem cell exhaustion [33, 108, 132]. The end result of these processes is an environment that promotes low-grade inflammation, along with decreased lymphoid progenitors and an accumulation of senescent lymphocytes with less diverse T cell repertoires [133–136]. Unlike lymphoid progenitors, myeloid progenitors are not diminished, which may contribute to increased incidence of anemias and myeloid dysplasias [137, 138]. Damage to critical hematopoietic stem cell niches and lymphoid microenvironments by HIV and ART may promote some HIV-associated comorbidities, such as younger onset or more aggressive presentation of some malignancies. HIV infection exacerbates these parameters, adding additional un-characterized physiological stressors that may not be fully resolved by ART, and creating a scenario in which biological aging could be accentuated or accelerated [108]. It will be important to identify lifestyle, behavioral, and environmental factors that modify the epigenetic and inflammatory background in treated HIV and determine whether their impact on morbidities and mortality is different in PWH vs uninfected adults. Understanding the senescent cells and SASPs that are most directly linked to chronic inflammation and comorbid conditions in older PWH may also help to identify pathways that could serve as anti-inflammatory therapeutic targets.

### Potential therapeutic targets to improve “healthspan” in older PWH

Understanding how HIV and ART affect physiological and health deficits associated with aging and exacerbate age-associated comorbidities may be key to identifying potential therapeutic targets that improve “healthspan” (ie, longer healthy life without significant comorbidities). A new therapeutic area is that of senotherapeutics for HIV and aging (reviewed in [139]). The demonstration in murine aging models that targeting of senescent cells can result in increased healthspan and lifespan [140] has prompted a variety of pharmacologic strategies to kill senescent cells (senolytic agents) or counteract senescence (reviewed in [139]). This rapidly developing field holds the promise of identifying compounds that target processes underlying cellular senescence and perhaps slowing aging-associated morbidities [141]. The first human trial of senolytics in patients with idiopathic pulmonary fibrosis (NCT02874989) investigated dasatinib (a tyrosine kinase inhibitor or TKI) in combination with quercetin (a plant flavanol that targets BCL-2, insulin/IGF-1, and HIF1-alpha), demonstrating the safety and tolerability of these compounds in association with improved physical function [142]. The role of these agents in the setting of HIV infection is not established and should be explored cautiously. Notable senolytics include BCL-2 antagonists, metformin, mTOR inhibitors, and Jak-1 and 2 inhibitors. The BCL-2 inhibitor venetoclax is being investigated for its role as a senolytic, and for its influence on HIV reservoirs. In animal models of aging, the efficacy of metformin led to the Targeting Aging with Metformin (TAME) Trial (managed by the American Foundation for Aging Research), investigating this drug as a novel senotherapeutic in HIV-uninfected persons. A small study (NCT02659306) with this compound [143] is being conducted in Canada in PWH to test whether its anti-inflammatory activity can reduce HIV reservoirs. Another interesting pharmacologic agent is the mTOR inhibitor rapamycin, which has been shown to prolong the lifespan of mammalian species [144], possibly due in part to improved effector cell responses. Everolimus, a rapamycin analog, administered with the seasonal influenza vaccine, improved vaccine responses in elderly participants [145]. Ruxolitinib, a Janus kinase (JAK) 1 and 2 inhibitor (FDA-approved for treatment of myeloproliferative disorders) has been investigated in a randomized phase 2 clinical trial in 60 PWH on ART (NCT02475655) and results of this trial are awaited.

Humans and NHPs on long-term ART can be studied to address many of these questions using newer technologies such as high-throughput omics platforms, assays for measuring mtDNA copy number and mitochondrial genetic variability, and multiplexed assays for quantifying inflammatory biomarkers that have expanded our ability to undertake a comprehensive integrated approach. Humanized mice and organoid models are also likely to be valuable in elucidating the effects of HIV and ART on various organs and tissues at the cellular and molecular level. Studies in the more closely genetically related NHP models that can be successfully treated with ART and develop SIV-related comorbidities [146] are also valuable to decipher pathways responsible for accentuated aging and end-organ disease. While studies on NHPs have translational potential for disease-related research [147], studies on aging with HIV/SIV have been limited to comparing infections of young and old animals [148, 149]. Epigenetic and other aging biomarkers described above can be incorporated into these studies to fill gaps in our scientific knowledge about biological aging and perhaps identify new approaches to improve healthy aging in PWH. To improve the lives of PWH, it will be important to determine which markers of accentuated aging are epiphenomena that might be useful markers of outcome and which contribute to pathogenesis that could serve as targets for intervention.

## CONCLUSIONS

We have reviewed evidence suggesting that people with HIV experience accentuated biological aging that is reflected by increased risk of many age-associated comorbidities, frailty, early mortality, persistent inflammatory states, and epigenetic markers of aging. HIV-specific factors that may influence biological aging in PWH on long-term ART include persistent low-level viral expression, increased gut permeability and dysbiosis of the microbiome/virome, direct effects of some ART drugs, and coinfections such as CMV and HCV. Moving forward, basic research, together with studies in NHP and small animal models, will help to develop a more complete mechanistic understanding of aging phenotypes that are affected by HIV and ART and make use of this knowledge to discover new targets for prevention and therapeutic intervention.
